# Personality Types of Medical Students in Terms of Their Choice of Medical Specialty: Cross-Sectional Study

**DOI:** 10.2196/60223

**Published:** 2024-12-31

**Authors:** Małgorzata Tobiaszewska, Tytus Koweszko, Jonasz Jurek, Karolina Mikołap, Jacek Gierus, Jantoni Mikulski, Napoleon Waszkiewicz

**Affiliations:** 1 Department of Psychiatry Faculty of Health Sciences Medical University of Warsaw Warsaw Poland; 2 Department of Environmental Psychiatry Faculty of Health Sciences Medical University of Warsaw Warsaw Poland; 3 JGResearch Warsaw Poland; 4 Department of Psychiatry Medical University of Bialystok Białystok Poland

**Keywords:** personality type, characteristics, traits, psychology, psychometric, medical students, university, burnout, gender, preferences, professional, job, career, specialty, survey, questionnaire

## Abstract

**Background:**

Research on personality types among doctors reveals its impact on medical specialty choices, suggesting that considering personality in career planning may enhance work satisfaction and reduce burnout risks.

**Objective:**

This study, encompassing 2104 medical students, explores how personality types, traits, and gender relate to specialty preferences.

**Methods:**

Participants of this study were medical students from various universities in Poland. The study surveyed 2104 participants. Each participant completed a general questionnaire and a NERIS Type Explorer personality test, based on the Myers-Briggs Type Indicator inventory and the “Big Five” personality traits concept. The questionnaire was distributed on social media groups for medical students from all Polish universities. An exploratory statistical analysis was performed to find relationships. For each tested relationship a Fisher exact test was conducted and the significance level was *P*<.05. Each test resulted in a *P* value and odds ratio (OR) with a CI. To ensure we included undecided students and obtained meaningful data, we allowed participants to select up to three medical specialties from the 77 available in Poland at the time of the study.

**Results:**

The findings unveil significant relationships between gender, personality types, traits, and specialty preferences. Women tended to favor Neonatology (OR 9.15, 95% CI 3.02-45.46), while men leaned toward Orthopedics and traumatology of the locomotor system (OR 7.53, 95% CI 4.87-11.94). Extroverted, Intuitive, Feeling, Prospecting, and Turbulent students showed a heightened interest in Psychiatry (OR 2.23, 95% CI 1.64-3.01), whereas Introverted, Observant, Feeling, Judging, and Turbulent types favored Family Medicine (OR 2.98, 95% CI 2.08-4.24) and Pediatrics (OR 2.13, 95% CI 1.51-2.99).

**Conclusions:**

In conclusion, this research establishes a link between personality and medical specialty selection. Taking into account the significant role of personality traits, it should be considered to integrate them into the process of selecting a medical career or designing a medical curriculum. This approach may allow for the customization of programs to match students’ traits, thereby cultivating improved clinical communication skills, fostering interprofessional collaboration and ultimately enhancing treatment outcomes and professional fulfillment among physicians. The main limitation of this study is that it was conducted on medical students, who lack the full knowledge of the work as a specific specialist. A study surveying medical doctors with longer internships across different wards could be conducted to check for any variabilities. Moreover, there are other significant factors that influence one’s medical specialty choice. Certainly, this area could be further explored.

## Introduction

Personality traits affect many significant life choices including career path. It was proven that a good match between the personality traits of an individual and those of the work environment has a positive effect on job satisfaction [1]. For instance, people with low levels of neuroticism will be more satisfied in occupations where there is a lower mean level of neuroticism, such as careers in science or technology, skilled, and managerial occupations [1]. The same is true for openness—the greater the fit between a worker’s personality and the average personality of the occupation, the bigger job satisfaction [1].

The above study’s results strongly correspond with two major theories of career choice. The first theory is the attraction-selection-attrition (ASA) model presented by Schneider [2]. The ASA model claims that people are attracted to, and selected by, specific environments according to their individual predispositions [2]. This selection effect creates environments that are consistent with people with specific attributes, which leads to significant person-environment fit. Moreover, employees who do not fit sometimes leave, and people who remain in the occupation will be similar to each other, and therefore, create a more homogenous group than those who were attracted to the work environment in the beginning [2].

The second model is Holland’s Vocational Theory [3]. It states that people select themselves into an occupation environment that matches their vocational interests, which further leads to greater contentment and better performance. Holland’s theory declares that most people fit into one of 6 personality types: Realistic, Investigative, Artistic, Social, Enterprising, and Conventional. The 6 vocational types characterize both occupational interests and individual differences in interests. Holland asserts that people of the same personality type when working together, create an environment that is best suited to them and their working style. The conclusion is that working among people with similar personality types brings more satisfaction and success at work [3].

The above results suggest that considering these career choice theories, choosing a medical specialty according to one’s personality type might lead to greater job satisfaction.

Existing research has demonstrated the applicability of vocational theories to the medical field. A study by Petrides and McManus [4] discovered strong similarities between Holland’s general career typology and the structure of medical careers, implying that the two frameworks are homologous. The research involved 3 large cohorts of medical students to generate maps of medical career paths. Medical specialties typical of Holland’s 6 RIASEC categories were Surgery (Realistic), Hospital Medicine (Investigative), Psychiatry (Artistic), Public Health (Social), Administrative Medicine (Enterprising), and Laboratory Medicine (Conventional) [4]. This homology between medical careers and RIASEC may mean that the map can be used as the basis for understanding career choice, and for providing career counseling [4].

Moreover, in their research, Borges et al [5] tested the hypothesis that medical specialties classified as technique-oriented or patient-oriented would be distinguished by the RIASEC code, with technique-oriented specialists resembling Investigative-Realistic types and patient-oriented specialists resembling Investigative-Social types. RIASEC code was consistent across both specialty groups, specifically Investigative-Social. The findings indicated that many medical students could be suited to a range of different specialties, suggesting that Holland’s model could help them assess how well their personalities align with various specialties and practice settings [5].

Apart from enhancing job satisfaction, improving the doctor-patient relationship is undoubtedly an important matter. One study showed that only around 11% of the medical personnel and 15% of the general population believe that the current doctor-patient relationship is harmonious [6]. It has been shown that doctors’ personalities differ significantly from their potential patients [7]. A small study of 313 medical graduates from the United Kingdom found that, compared to the general adult population, doctors had a preference for Introversion over Extraversion, Intuitive Perception over Sensing Perception, Thinking Judgment over Feeling Judgment, and a Judging orientation over a Prospecting orientation [7]. It has been shown that training can help alleviate the challenges posed by personality differences, leading to better interaction outcomes [8]. A recent study suggests that communication training, based on students’ personality traits and aimed toward improving the doctor-patient relationship should be implemented in medical studies curriculum [9].

Furthermore, existing research confirmed that empathy is related to personality and that it can be improved and taught [10,11]. Medical empathy is defined as primarily the cognitive ability to understand patients’ experiences, concerns, and perspectives, and to communicate this understanding with the intent to help [12,13]. Different personality factors can be integrated into medical education to enhance empathy, with pedagogical objectives focused on empathy development [10]. Individualized intervention strategies based on personality traits should be comprehensive, considering the complexity of empathy [10,11]. Moreover, personality preference research has contributed to a better understanding of teaching styles and clinical performance test achievements [14,15]. These results highlight the importance of exploring the personality traits of medical students and doctors in order to develop effective training programs aimed at enhancing empathy. Such programs could also serve as a foundation for research on how this training impacts the doctor-patient relationship.

Beyond improving empathy and the doctor-patient relationship, it is also crucial to prioritize physicians’ mental health. In Poland, burnout affects as many as 67% of physicians [16]. The literature demonstrates that certain personality types, especially Realistic types, are more prone to burnout than, for example, Social or Artistic personality [17]. Regarding medical specialties, burnout prevalence is notably higher in surgical specialties compared to clinical specializations [18]. Students who are aware of their personality traits might make more informed decisions when selecting between these fields.

Up to now, no research has been done on Polish medical students that would so extensively investigate the influence of personality type on the choice of a medical career. This paper is notable because it surveyed a large number of students questioned (around 6% of all medical students in Poland [19]) and considered various factors including gender, academic year, personality type, and personality traits. Furthermore, it facilitates the comparison of the data with existing research on the subject. The authors analyzed the link between demographic factors and personality traits exhibited by medical students and their choice of specialties. Such a thorough examination allows for better comprehension of choices regarding medical specialties made by Polish students. This may benefit in raising awareness about burnout prevention and job satisfaction.

This study aimed to determine whether personality type affects Polish medical students’ career choices.

## Methods

### Study Design

This cross-sectional study was conducted in March 2020. Each participant answered a questionnaire that included both the NERIS Type Explorer, as well as other questions regarding demographics and the chosen medical specialty. The questionnaire was distributed on social media groups for medical students from all Polish universities and it was available for 1 week from March 16, 2020, to March 23, 2020. It was completely anonymous and all participants took part in the digital survey out of their own free will, using their own personal devices, such as smartphones or PCs. The only inclusion criterion was being a medical student. Participants, who admitted they were not medical students were omitted in the analysis. Not to exclude undecided students from our study and to receive as meaningful data as possible, we asked for the choice of up to three medical specialties from 77 available in Poland at the moment of the study.

The data collected in the questionnaires were analyzed using NERIS Type Explorer to obtain each participant’s personality type. We analyzed each respondent’s personality type and the intensity of a specific personality trait, measured on a scale from –100 to +100, as seen in the Multimedia Appendix 1. Surveys were collected using a web-based tool Typeform (Typeform S.L.) [20].

### Statistical Analysis

An exploratory statistical analysis, descriptives, crosstables, and logistic regression were performed to find associations between the personality type, personality traits, chosen specialty, and demographic data. The study was designed to obtain pilot data from Polish students. For each tested association a Fisher exact test was conducted since this test is fairly resilient to variations in sample characteristics. However, in this case, these characteristics are not well controlled. The significance level was *P*<.05. Each test resulted in a *P* value and odds ratio (OR) with a CI. For a test to be conducted, the following inclusion criteria had to be met: for each subgroup in the contingency table, there had to be at least one person in the subgroup and the number of expected frequencies for each subgroup had to be at least 5. Scatter plots were used to visualize data. The required sample size to achieve statistically significant effects at an α significance level of 5%, assuming a beta sensitivity of 80% in the Fisher exact test for an effect size with an OR in the range of (0.75-1.5), considering Bonferroni correction for multiple comparisons across a family of related hypotheses, and assuming a response accuracy rate of >95%, was 2108.

To account for the multiple comparisons problem, we applied the Bonferroni correction for the cutoff point of the significance level. A primary limitation in multiple comparisons is that as the number of dimensions compared increases (which is considerable in this study), the mathematical probability of obtaining statistically significant results also increases. This probability is present here, necessitating a more cautious interpretation of the findings. To estimate the family wise error rate, the associations were divided into a few families (personality type vs specialty, personality type vs gender etc). The cutoff value for recognizing a *P* value as significant, which ranged from 0.003 to 0.02 depending on the number of tests conducted, was determined by dividing the significance level (5%) by the number of tests within a family.

All the computations were performed using R software (version 4.1.2; R Core Team, R Foundation for Statistical Computing). The packages used were stats, dplyr, and ggplot2.

### Instrument for Data Collection

Myers-Briggs Type Indicator (MBTI) is a personality inventory created by Isabel Briggs Myers and Katherine Briggs and based on the theory described by Carl Gustav Jung [21-23]. It identifies 4 personality types and later divides each of them into 4 more subtypes, distinguishing overall 16 personality types. The name of the type consists of 4 letters, describing certain personality traits: E or I (for Extraversion or Introversion, understood as the preference for the outer or inner world), S or N (for Sensing or Intuition, understood as the preference for relying on the basic information or interpreting and adding meaning), T or F (for Thinking or Feeling, understood as the preference for logical thinking or seeing special circumstances), and J or P (for Judging or Prospecting, understood as the preference for making strong decisions or being open to new options). The MTBI instrument has been validated, used in various studies over the past 40 years, and described as reliable and accurate [21-23].

NERIS Type Explorer is an instrument used by the creators of the website 16personalities [24]. It is based on the MBTI inventory, as well as on the “Big Five'' personality traits concept [24]. In addition to using 16 types identical to the Myers-Briggs test, it allows to add another aspect to the classic 4-letter personality type of the MTBI inventory, using either -A or -T (for Assertive or Turbulent, understood as how confident people are in their decisions and abilities). In consequence, it consists of 32 separate subtypes all of which are presented in Multimedia Appendix 2.

### Ethical Considerations

The study was conducted in accordance with the Declaration of Helsinki and was approved by the ethics committee of the Medical University of Warsaw (AKBE/62/2023, March 06, 2023). Participation in the questionnaire was voluntary and data acquired from it were completely anonymous.

## Results

### Characteristics of the Study Group

In 2020 there were approximately 37,000 medical students studying at 22 different Polish medical universities [19]. Our questionnaire was filled out by 2104 medical students from Poland.

Among 2104 students who filled in our questionnaire, the majority (n=1645, 78%) of people were female, while only 454 (22%) were male. Five students chose the “other” option in the gender-related question. A total of 1830 (86%) surveyed students declared heterosexual orientation, 141 (6%) students as bisexual, 84 (4%) students as homosexual, 16 (0.4%) students as asexual, and 33 (1.6%) students chose the option “other or refuse to answer.”

The mean age of our surveyed population was 23 (SD 2.150) years, with the age range from 18 to 40 years.

In Poland, medical studies last 6 years and clinical rotations start during the third year. Students participating in our survey represented all 6 years of studies, however, at the moment of the survey, most students were in their third or fourth year. Consequently, around 3/4 of our surveyed population had started classes in the hospital, encountered patients, and by this time could already have an opinion on the type of clinical work they enjoy or value the most.

In total, 99.6% of the students questioned in our study came from public-funded Polish universities.

### Most Common Personality Types

We examined what were the most common personality types in our study population. Using the division into 16personalities, the most common types were: ESFJ (Extroverted, Observant, Feeling, Judging; n=280, 13.3%), ISFJ (Introverted, Observant, Feeling, Judging; n=258, 12.3%), ENFJ (Extroverted, Intuitive, Feeling, Judging; n=233, 11.1%), ENFP (Extroverted, Intuitive, Feeling, Prospecting; n=210, 9.9%), and INFJ (Introverted, Intuitive, Feeling, Judging; n=204, 9.7%). These 5 most popular personality types were true for more than half of our study group. After differentiation into Assertive and Turbulent subtypes, within 32 possible variants, 5 most popular were: ISFJ-T (Introverted, Observant, Feeling, Judging, Turbulent; n=187, 8.9%), INFJ-T (Introverted, Intuitive, Feeling, Judging, Turbulent; n=160, 7.6%), ESFJ-A (Extroverted, Observant, Feeling, Judging, Assertive; n=144, 6.8%), ENFJ-T (Extroverted, Intuitive, Feeling, Judging, Turbulent; n=143, 6.8%), and ENFP-T (Extroverted, Intuitive, Feeling, Prospecting, Turbulent; n=136, 6.5%). It should be noted that there was a vast disproportion between the prevalence of the most and least common types and subtypes, as we discovered only 31 (1.5%) cases of the ISTP (Introverted, Observant, Thinking, Prospecting) type and only 9 (0.4%) of the ISFP-A (Introverted, Observant, Feeling, Prospecting, Assertive) subtype. Table 1 shows the number and percentage of participants presenting specific personality traits.

**Table 1:** Number and percentage of participants presenting Extroverted or Introverted (E/I), Intuitive or Observant (I/O), Thinking or Feeling (T/F), and Judging or Prospecting (J/P) traits in our study group.

Trait		Value (N=2104), n (%)
**E/I**		
	Extroverted^a^	1142 (54.3)
	Introverted	962 (45.7)
**N/S**		
	Intuitive^a^	1112 (52.8)
	Observant	992 (47.2)
**T/F**		
	Thinking	638 (30.3)
	Feeling^a^	1466 (69.7)
**J/P**		
	Judging^a^	1403 (66.7)
	Prospecting	701 (33.3)

^a^The trait with a higher prevalence in each pair.

Interestingly, personality type that combined all of the above predominating features (ENFJ) was only the third most common type in our population. It shows how the prevalence of certain traits is not always reflected in the prevalence of one of 16 personality types that present all of these individual traits at once.

### Most Common Specialties

Among 77 medical specialties available in Poland, the ones that were chosen the most often in our survey were Pediatrics (n=393, 18.68%), Psychiatry (n=325, 15.45%), Family Medicine (n=294, 13.97%), Anesthesiology and Intensive Care (n=284, 13.50%), Endocrinology (n=282, 13.40%), Internal Medicine (n=264, 12.55%), Cardiology (n=254, 12.07%), Obstetrics and Gynecology (n=237, 11.26%), Dermatology and Venereology (n=216, 10.27%), and Radiology and Imaging Diagnostics (n=200, 9.51%).

### Gender Differences

Men more often belonged to the Thinking group (OR 3.18, 95% CI 2.55-3.98), while women were more dominant in the Turbulent group (OR 2.85, 95% CI 2.3-3.55). Moreover, men more frequently were INTJ-A (Introverted, Intuitive, Thinking, Judging, Assertive; OR 7.16, 95% CI 3.45-15.52) and ISTJ-A (Introverted, Observant, Thinking, Judging, Assertive; OR 3.87, 95% CI 2.1-7.14). They were also more abundant in ESTP (Extroverted, Observant, Thinking, Prospecting; OR 4.22, 95% CI 2.12-8.44), INTP (Introverted, Intuitive, Thinking, Prospecting; OR 3.83, 95% CI 2.24-6.54), and INTJ (Introverted, Intuitive, Thinking, Judging; OR 2.7, 95% CI 1.7-4.23) groups regardless of A/T feature. The women group was characterized by greater presence of ISFJ-T (Introverted, Observant, Feeling, Judging, Turbulent; OR 2.95, 95% CI 1.74-5.34) and ENFP-T (Extroverted, Intuitive, Feeling, Prospecting, Turbulent; OR 2.28, 95% CI 1.53-3.5) personality types. The strongest association between gender and preference of medical specialty was observed in Neonatology, which was chosen over 9 more times by women (OR 9.15, 95% CI 3.02-45.46), and Orthopedics and traumatology of locomotor system—chosen 7 times more by men (OR 7.53, 95% CI 4.87-11.94). Moreover, women were more commonly interested in Obstetrics and Gynecology (OR 4.2, 95% CI 2.5-7.56), Pediatrics (OR 2.82, 95% CI 1.99-4.08), and Dermatology and Venereology (OR 2.78, 95% CI 1.75-4.65). Men were more inclined to choose Urology (OR 5.4, 95% CI 3.11-9.48), Cardiology (OR 2.08, 95% CI 1.55-2.97), and Anesthesiology and Intensive Care (OR 1.96, 95% CI 1.48-2.59). Generally, women were more drawn to choose pediatric specialties (OR 3.33, 95% CI 2.47-4.51). On the other hand, men preferred specialties related to surgeries (OR 2.76, 95% CI 2.2-3.48). Table 2 shows the number and percentage of participants reporting interest in specialties depending on gender.

**Figure 1:**
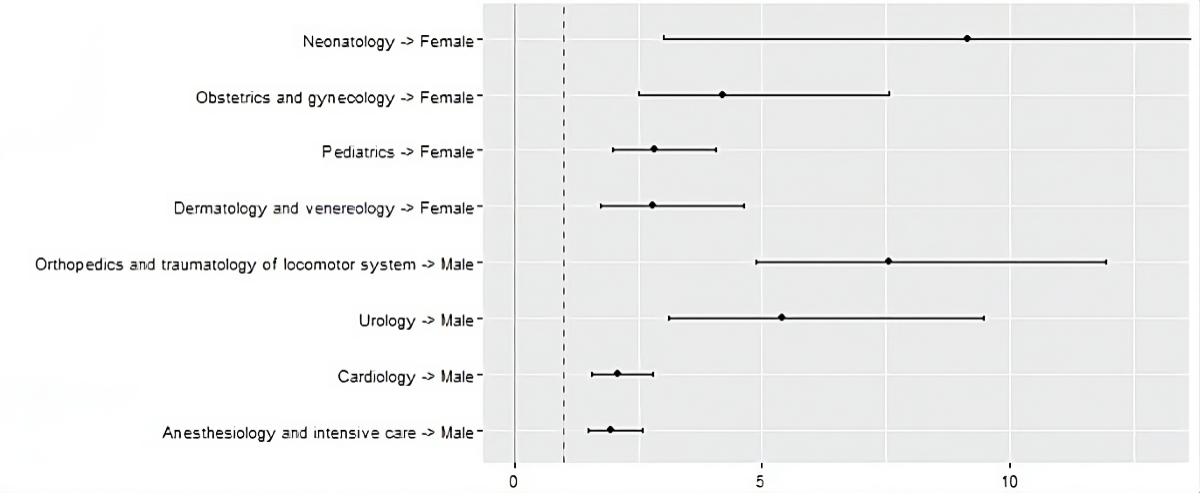
The association between medical specialty choice and gender, presented as odds ratios with 95% CIs.

The association between medical specialty choice and gender, presented as ORs with 95% CIs, is shown in Figure 1.

**Table 2:** Number and percentage of participants reporting interest in specialties depending on gender.

Specialty	Women, n (%)	Men, n (%)
Neonatology	96 (5.84)	3 (0.66)
Orthopedics and traumatology of locomotor system	35 (2.13)	65 (7.71)
Obstetrics and gynecology	218 (13.25)	17 (3.74)
Pediatrics	355 (21.58)	38 (8.37)
Dermatology and venereology	196 (11.91)	20 (4.41)
Urology	23 (1.4)	35 (7.71)
Cardiology	170 (10.33)	83 (18.28)
Anesthesiology and intensive care	194 (11.79)	90 (19.82)

### Personality Traits

**Figure 2:**
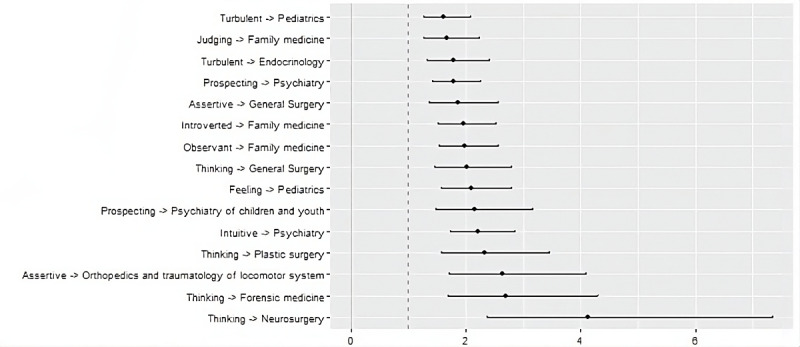
The association between the chosen medical specialty and personality trait, presented as odds ratios with 95% CIs.

Family Medicine was chosen almost two times more often by Introvert (OR 1.96, 95% CI 1.52-2.53), Observant (OR 1.98, 95% CI 1.54-2.56), or Judging (OR 1.68, 95% CI 1.27-2.24). Being Intuitive (OR 2.22, 95% CI 1.73-2.86) and Prospective (OR 1.79, 95% CI 1.41-2.26) are associated with interest in Psychiatry. Moreover, Prospective students were also keen on the Psychiatry of Children and Youth (OR 2.16, 95% CI 1.48-3.17). Thinking trait was more common in the group of students choosing Neurosurgery (OR 4.14, 95% CI 2.37-7.34), Forensic Medicine (OR 2.7, 95% CI 1.69-4.31), Plastic Surgery (OR 2.33, 95% CI 1.57-3.45), and General Surgery (OR 2.02, 95% CI 1.46-2.79) while Feeling was more popular in Pediatrics (OR 2.09, 95% CI 1.58-2.8). Overall, Thinking went with specialties involving surgeries (OR 1.69, 95% CI 1.39-2.06). Feeling was associated with pediatric specialties (OR 1.84, 95% CI 1.46-2.32). Respondents with Assertive trait were more commonly picking surgical specialties (OR 1.64, 95% CI 1.37-1.97), especially Orthopedics and traumatology of locomotor system (OR 2.64, 95% CI 1.71-4.08) and General Surgery (OR 1.87, 95% CI 1.36-2.57). In contrast, the Turbulent trait favored specialties of Internal Medicine (OR 1.42, 95% CI 1.17-1.71). Additionally, Turbulent also favored Pediatrics (OR 1.62, 95% CI 1.27-2.08) and Endocrinology (OR 1.78, 95% CI 1.33-2.41). The association between the chosen medical specialty and personality trait presented as ORs with 95% CIs, is shown in Figure 2.

### Personality Type

**Figure 3:**
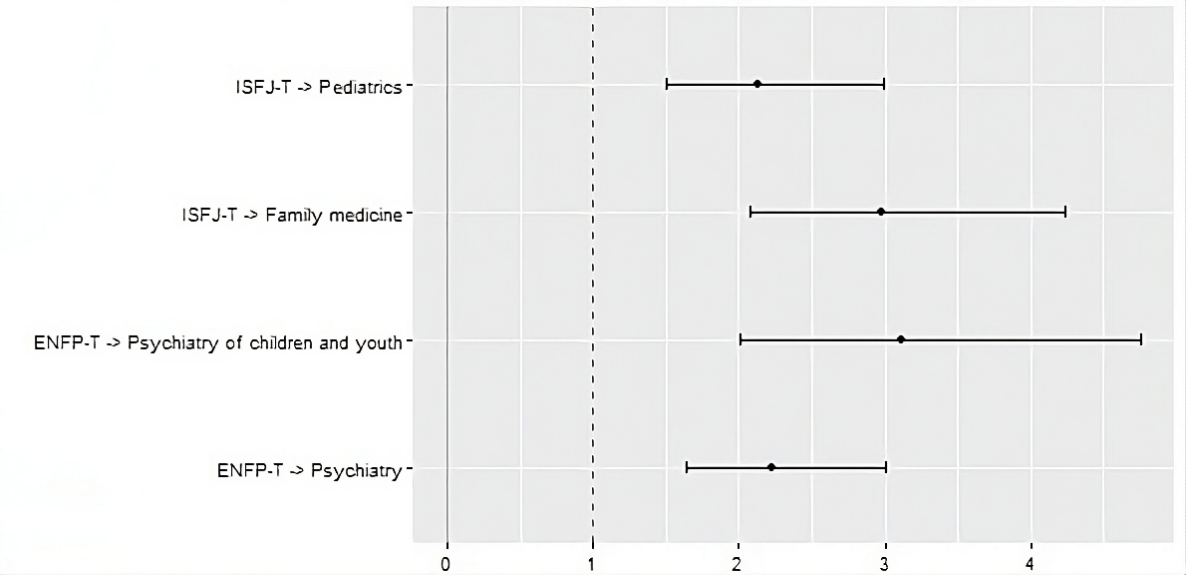
The association between the chosen medical specialty and personality type, presented as odds ratios with 95% CIs.

Respondents with ENFP-T personality type seemed to be interested in Psychiatry (OR 2.23, 95% CI 1.64-3.01) and Psychiatry of Children and Youth (OR 3.11, 95% CI 2-4.75). Moreover, ESTJ-A were often choosing specialties related to surgeries (OR 2.61, 95% CI 1.57-4.5). ISFJ-T personality type was negatively associated with interest in surgical specialties (OR 0.32, 95% CI 0.18-0.53), but positively with Family Medicine (OR 2.98, 95% CI 2.08-4.24) and Pediatrics (OR 2.13, 95% CI 1.51-2.99). INTJ-A very rarely chose pediatric specialties, choosing them over 16 times less than the rest of the respondents (OR 0.06, 95% CI 0.002-0.385). The association between chosen medical specialty and personality type presented as ORs with 95% CIs, is shown in Figure 3.

### Specialties, Which Associate Each Other

Students who chose Anesthesiology and Intensive Care often chose Emergency Medicine (OR 4.51, 95% CI 2.6-7.71). General Surgery was often picked together with Oncological Surgery (OR 9.26, 95% CI 5.22-16.23) and Pediatric Surgery (OR 4, 95% CI 2.23-6.92). Internal Medicine was associated with Gastroenterology (OR 3.28, 95% CI 1.98-5.29). Endocrinology was commonly cochosen with Gynecological Endocrinology with reproductiveness (OR 3.82, 95% CI 2.13-6.7), which was also common among students, who picked Obstetrics and Gynecology (OR 5.61, 95% CI 3.22-9.91). Just like Pediatrics and Neonatology (OR 4.46, 95% CI 2.93-6.78), Pediatrics and Pediatric Gastroenterology together were a common founding (OR 12.76, 95% CI 5.14-35.99). Specialties, that were very common among students interested in Psychiatry, were Psychiatry of Children and Youth (OR 8.15, 95% CI 5.51-12.11) and Sexology (OR 5.08, 95% CI 2.54-10.15).

**Figure 4:**
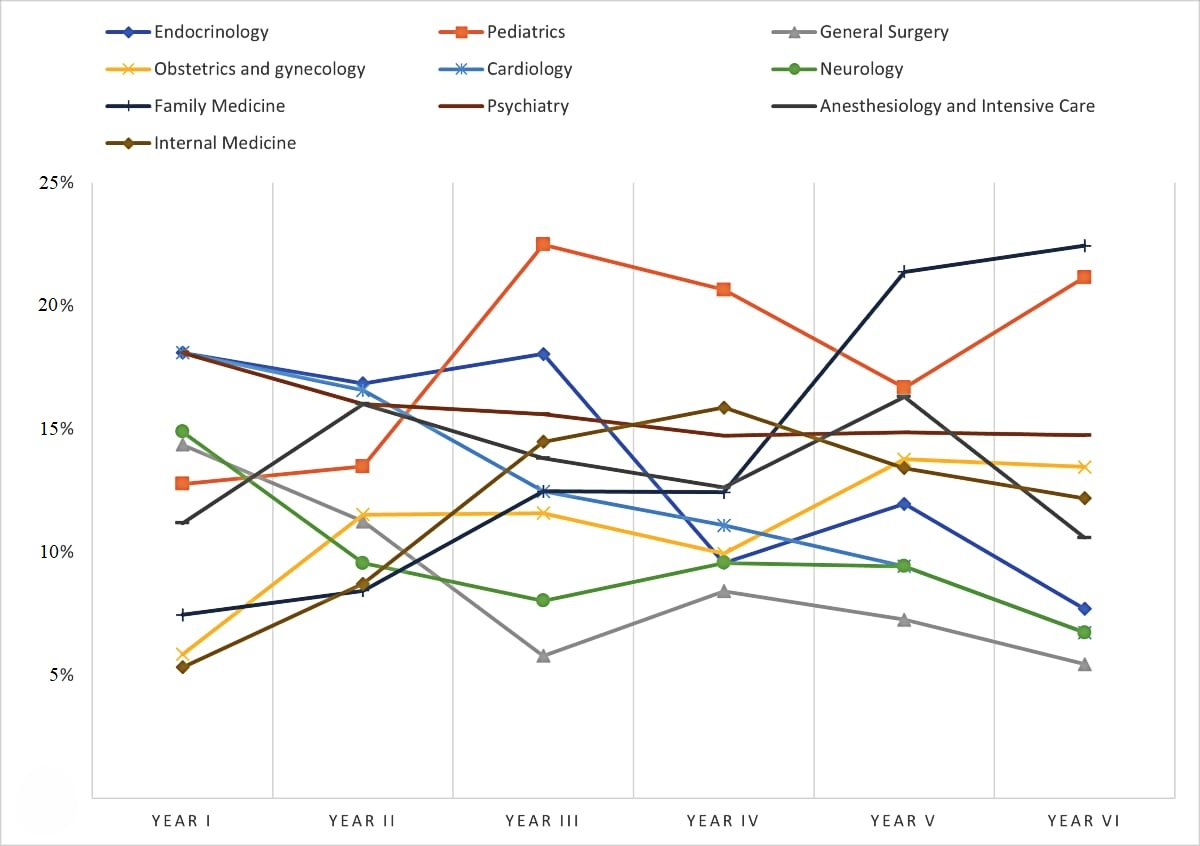
Ten frequently selected medical specialties based on the year of study.

Figure 4 illustrates the ten most frequently selected medical specialties based on the year of study.

## Discussion

### Principal Findings

In this research, we aimed to find relationships between personality types defined by NERIS Type Explorer and future specialty preferences among the Polish medical school population. The analysis showed that Extraverted, Intuitive, Feeling, and Prospecting (ENFP) personality, as well as Intuitive and Prospecting assets in general, are more common in students interested in Psychiatry. Moreover, the Thinking trait and ESTJ-A (Extraverted, Observant, Thinking, Judging, Assertive) personality type related positively with the choice of specialties that involved surgeries. Students who were classified as Introverted, Observant, and Judging or had ISFJ-T personality were more likely to choose Family Medicine.

Our findings are consistent with those of previous research conducted on American medical students [25]. A longitudinal study that collected data from both students and graduates showed a statistically significant relationship between the Extroversion-Introversion (E-I) and Thinking-Feeling (T-F) axes and the choice between primary and nonprimary specialties [25]. According to the authors, Extraverted and Thinking types were more inclined toward surgical professions whereas Introverted and Feeling types are more likely to favor primary care [25]. Another research sought for the relationships between the MBTI type assigned in the first year of school and the actual specialty choice in the postgraduate year [26]. Three associations were found statistically significant. The interviewees who were classified as Sensing, Feeling, and Judging type tended to choose Family Medicine. Sensing, Thinking, and Judging types were also prone to follow a career path in Obstetrics-Gynecology. Last but not least, Psychiatry was preferred by Intuitive, Feeling, and Prospecting types [26]. As far as particular MBTI personality types are concerned, our findings are consistent with data adapted from Freeman [27] and McCaulley [28], which indicates that Feeling and Intuitive types tend to prefer Psychiatry, while Sensing, Thinking, and Judging (STJ) types are more inclined toward Surgery.

Similar studies were performed in veterinary medicine students, but their results differ significantly. In the study describing students of the Veterinary Faculty in Slovenia, more students presented Introverted, Sensing, Thinking, and Judging traits, and the most common type was ISTJ [29]. These findings are very different than in this study’s results, as in our population, most medical students had Extroverted, Intuitive, Feeling, and Judging traits and ISTJ type was only the eighth most common personality type. The authors of the discussed veterinary students study suggested that veterinarians are often more caring, have high levels of compassion, and choose their careers because of their social responsibilities awareness [29]. By contrast, one might wonder if traits more common in medical students could be more associated with the need for contact with other people (Extroversion) and compassion for patients experiencing suffering (Feeling). Certainly, this topic needs further research.

Another veterinary student’s research regarding MBTI types was performed in Canada and showed predominant traits to be Extraversion, Sensing, Thinking, and Judging [30]. Two of the most prevalent MBTI types in the overall sample were ESTJ and ISTJ, respectively, the seventh and eighth most common types in our group [30]. This study is particularly interesting because it compared factors such as MBTI types, gender, and career preferences in a similar way as this work. Some findings are parallel to our results: for example, the odds of preferring Thinking rather than Feeling were approximately three times greater for male veterinary students compared to female students, which is consistent with our OR of 3.18 for men belonging to the Thinking group. In this Canadian work, the only statistically significant association between gender and desired specialty was surgery with an OR of 1.48 [30]. The authors found differences in the preferred career path and personality traits, that is, students were more likely to select equine practice for their final-year stream if they exhibited the Prospecting trait versus Judging [30]. Furthermore, students from the Thinking group were more likely to consider herd health management, reproduction, or pursuing a career in the government [30]. It was suggested that these fields, with the logical and impersonal preference of Thinking, are more appealing to such persons [30]. The above results show that veterinary school students had different personality traits and types than our group of medical students and that there are important influences of gender and personality on the choice of the future career path in veterinary students, as well as in medical students.

What is more, previous studies have shown that certain personality traits appear more often in physicians of some medical specialties. For instance, physicians working in Emergency.

Medicine was more likely to be extroverted [31]. It has also been proven that surgical specializations are much more often chosen by people with a personality preference for Thinking rather than Feeling [25]. What is interesting is the matter of why certain personality traits relate to specific specialty preferences.

The website 16 personalities offers users a detailed explanation of various personality traits and types, which can be determined through the personality test [24]. For example, Extroverts are described as being interested in engaging with their environment and feeding off the responses of the people and events around them, which may help them manage stress in specialties requiring frequent interpersonal interactions or unpredictable situations [32]. This corresponds with our findings, where Psychiatry—characterized by extensive interpersonal interaction—or Surgery, which can involve unexpected procedures, were more commonly chosen by Extroverted individuals. Introverts, by contrast, are characterized by a preference for solitary environments where they can regulate their surroundings and engage in focused “alone time,” potentially leading to deeper insights [32]. This indicates that Introverts may experience lower stress in stable environments with fewer new interactions. Our finding that Introverted types more often choose specialties like Family Medicine or Pediatrics, where patients are typically familiar to the physician, aligns with this observation.

The idea behind our study is concern about preventing burnout among the population of medical doctors by finding the best fit between one’s personality type and a medical specialty. In the *ICD-11* (*International Statistical Classification of Diseases and Related Health Problems, 11th Revision*), burnout is classified as an occupational phenomenon, characterized by 3 dimensions: “feelings of energy depletion or exhaustion; increased mental distance from one’s job, or feelings of negativism or cynicism related to one’s job; and reduced professional efficacy” [33]. The concept of making occupational choices based on personality traits has been present in psychological discourse for decades, resulting in many inspiring works. One of them is Holland’s Theory of Career Choice (RIASEC), which links one’s key skills and interest areas to certain occupations and subjects [3]. This approach, which Kristof-Brown calls person-vocation fit, seems to be a suitable theoretical framework in terms of matching MBTI personality types with particular medical specialties [34].

To establish a connection between personality traits, as well as problem-solving approaches, and the RIASEC code, Ackerman’s [35] and Ackerman and Heggestad’s [36] work provide valuable insights. Ackerman [35] and Ackerman and Heggestad [36] demonstrated that measures of intellectual ability predominantly relate to interests in Realistic, Investigative, and Artistic careers. Individuals with higher verbal abilities tend to prefer Artistic and Investigative careers, while those with stronger spatial and mathematical abilities are more inclined toward Realistic and Investigative careers [35,36]. In terms of personality measures, Ackerman found relationships with the SEC components of the RIASEC code, using the Big Five personality typology for description [35,36]. Specifically, Extraversion is primarily correlated with an interest in Social and Enterprising careers, whereas Conscientiousness is linked to an interest in Conventional and Enterprising careers. Openness to Experience is somewhat a mixture of intellectual ability and personality and correlated positively to Artistic, Investigative, and Realistic careers, and negatively with Conventional careers [35,36]. Similar results were obtained by Larson et al [37], who conducted a meta-analysis, revealing significant correlations between Holland’s 6 vocational interest dimensions and the Big Five personality traits. For instance, they found strong correlations such as Artistic with Openness, Enterprising with Extraversion, Social with both Extraversion and Agreeableness, and Investigative with Openness [37].

By applying Holland’s Theory of Career Choice to our results, and following the approach of Petrides and McManus, we can align each element of the RIASEC code with a corresponding medical specialty [4]. Surgery is considered the best representation of the Realistic type, characterized by those who “enjoy activities requiring physical strength, aggressive action, motor coordination, and skill” [3,4]. Its heavy reliance on manual and technical skills at the operating table makes Surgery particularly fitting. In this study, individuals interested in Surgery predominantly exhibited Thinking and Assertive traits. According to 16personalities, those with the Thinking trait rely on objective information, testing alternatives against logic and reason to determine the most effective or realistic decision [38]. Assertive individuals are characterized as self-assured, even-tempered, and stress-resistant [39]. These descriptions may match Ackerman’s findings that intellectual ability and a logic-based approach to problem-solving correlate with Realistic careers [35,36].

Holland defines the “Investigative Type” as someone who prefers systematic and creative investigation of physical, biological, and cultural phenomena [3]. Petrides and McManus [4] identify doctors in Internal Medicine as Investigative types, given the necessity of a comprehensive, investigative approach, particularly in hospital settings. Our research found that the Turbulent trait is strongly associated with this specialty. 16personalities describes Turbulent individuals as success-driven, perfectionistic, and eager to improve, often addressing minor issues before they escalate [39]. This trait might align with the Investigative type’s inclination for in-depth analysis.

“Artistic Type” is defined by Holland as individuals with aesthetic interests who prefer indirect interactions and express themselves through artistic means [3]. Petrides and McManus [4] identified Psychiatry and General Practice (Family Medicine in our research) as fitting this description [4]. However, we chose to separate these specialties, associating Family Medicine with the Artistic Type and Psychiatry with the Social Type. This decision was based on the lack of significant associations between the Public Health specialty (representing the Social Type in Petrides and McManus) and personality in our research. Additionally, in the Petrides and McManus research, Psychiatry in the general population sample was still more concerned with People, which fits well with the reduction of Holland’s hexagon to the two dimensions of Things-People and Ideas-Data [3,4]. In our research, Family Medicine associates with the ISFJ-T personality, known for being sensitive, caring, analytical, and detail-oriented, with strong people skills despite their reserved nature [40]. This aligns with Ackerman’s depiction of Artistic careers, where people noted for their strong verbal and social abilities are most fitting [35,36].

Holland’s “Social Type” refers to individuals who prefer activities involving influencing others to inform, train, develop, cure, or enlighten [3]. As mentioned above, we identified Psychiatry as the most fitting specialty for this type, with our results showing an association between Psychiatry and the ENFP-T personality, known for being extraverted, outgoing, and seeking deep emotional connections [41]. These findings match with Ackerman’s research, which links extraversion to Social careers [35,36].

Holland describes the “Enterprising Type” as someone who has “a preference for actives that entail the manipulation of others to attain organization goals or economic gain...these behavioral tendencies lead in turn to an acquisition of leadership, interpersonal, and persuasive competencies” [3]. Petrides and McManus [4] suggest that Administrative Medicine is most suited to this type, though our research identified no equivalent specialty. Previous research shows the relationship between interest in Enterprising careers with Extraversion and Conscientiousness [35,36]. In the NERIS Type Explorer, the trait analogous to Conscientiousness is Judging, characterized by a strong work ethic and prioritization of duties [42]. Our results indicated that surgical specialties were associated with both Extraversion and Judging traits, aligning them closely with Holland’s Enterprising type [3].

Holland defines the “Conventional Type” as individuals who prefer systematic manipulation of data to achieve organizational goals. Petrides and McManus [4] have linked this type with Laboratory Medicine; however, our research found no significant association between Laboratory Medicine and personality traits or types. Interest in Conventional careers correlated with Conscientiousness, which, as mentioned above, may be represented by the Judging trait. Our findings revealed that the Judging trait was associated with Family Medicine and was also present in the ESTJ-A personality type, which is associated with surgical specialties, and the ISFJ-T personality type, linked to Family Medicine and Pediatrics. Thus, there is no clear link between Holland’s Conventional Type and specific medical specialty in our findings.

Although some skills, qualities, and requirements are to be found in a number of medical disciplines, some of these traits are exclusive to a certain specialty. For instance, the authors of a study conducted at Chang Gung Memorial Hospital observed that emergency physicians demonstrated less preference for Sensing trait than other attending physicians [43]. It was suggested that the Intuitive trait of the emergency physicians is a result of the circumstances of the work in the emergency department, where pattern recognition is crucial to diagnose conditions presenting ambiguous symptoms in a short time. A notable finding from the study is that while both junior doctors and attending physicians displayed similar personality types depending on their medical specialty, attending physicians exhibited more pronounced Sensing, Thinking, and Judging traits. The study’s authors suggest that clinical experience and an evidence-based approach contribute to this tendency [43]. However, according to the ASA model, organizational culture is shaped by three factors [2]: (1) individuals are attracted to organizations where members share similar personalities, values, interests, and attributes; (2) organizations tend to select individuals who possess knowledge, skills, and abilities similar to those of existing members; and (3) those who do not fit well are more likely to leave.

This raises the question: does the similarity among attending physicians result from the work environment—where individuals who are dissatisfied or unable to adapt leave—or from the environment imposing specific personality traits on people? Or maybe it is the interpersonal influence, where colleagues develop specific traits from each other? Or perhaps the combination of both? Subsequently, research that defines if having more distinct MBTI personality traits is related to job satisfaction would also be beneficial to early burnout prevalence.

Some personality traits are also crucial for satisfaction from the working conditions. Given the fact that our survey was distributed during the early weeks of the pandemic of SARS-CoV-2 when most Polish universities switched to a web-based–classes-only policy, it might have influenced the moods of certain students due to their preferences for the intensity of social life. Our group had a slight predominance of Extroverts. It was proven that university students with this trait tended to dislike web-based classes as they preferred meeting people and consulting with their lecturers face-to-face. They also had trouble focusing during such classes. In contrast, Introverts preferred staying at home and communicating by writing [44]. Perhaps such preference influences the perception of subjects that were taught digitally versus the ones that were taught in person. It would seem interesting to examine this topic in the future to assess whether the fact that different subjects were taught digitally or not at the university during the pandemic influenced the choice of medical specialty by Extroverted and Introverted medical students after their studies.

Our findings correspond with studies on populations from different cultural and socioeconomic backgrounds within contrasting health care systems. This suggests that a model similar to the Holland Occupational Themes, tailored to medical fields as proposed by Petrides and McManus [4], could be used to develop programs that help match students’ traits with the most suitable specialties. These programs could also be used to enhance students’ self-awareness, helping them to identify and develop their strengths while addressing weaknesses as they prepare for a career in their chosen medical specialty. Such programs should be considered as guidance rather than strict recommendations for student’s career choices, given that numerous factors could influence job satisfaction and performance. A recent study of medical students identified key influences on the decision to pursue a career in Orthopedics, including familial and peer pressure, perceptions of job prestige, gender considerations, parental involvement, elective rotations, opportunities for research and teaching, and aspirations for leadership roles [45]. Consistent with our findings, the study also identified an association between the choice of Orthopedics and male gender [45]. Another study on medical students and interns in Saudi Arabia found that factors influencing the choice of a medical specialty were interests in the procedures and techniques specific to the specialty, dissertation research experience, high-quality teaching within the program, connections with friends, relatives, or others in the health care field, and positive experiences with physician role models [46]. Additionally, work-life balance was found to be a significant consideration for students when selecting a specialty [47].

Nevertheless, exploring students’ personalities could also be used in cultivating and enhancing behaviors desirable in physicians, such as empathy. All of the above with the aim to improve treatment outcomes and professional satisfaction among physicians. Such initiatives could serve as a foundation for longitudinal research to examine whether this training impacts the doctor-patient relationship and burnout rates. Extensive sociopsychological research on the medical doctor population would be needed. Undoubtedly, this area is yet to be covered.

This study is noteworthy due to the extensive size of the respondent group surveyed while considering various factors such as gender, academic year, personality type, and personality traits. Additionally, it facilitates the comparison of the data with prior research in the field.

### Limitations

There are several limitations inherent in this study.

First, the major limitation that could be addressed in future research is that it was conducted on medical students, who, inevitably, do not possess the full picture of the work as a specialist in a specific area. Therefore, their declared choice of future specialty is based on a general impression that was formed during a relatively short time spent in particular wards.

Second, as Kristof Brown indicates, apart from the person-vocation fit, there are other significant factors that influence one’s job satisfaction, that is, person-job, person-organization, person-group, and person-supervisor fit [34]. Hence, even if a model linking personality type with medical discipline is created, the true impact on the individual level cannot be concluded.

Third, although some distinctive traits seem to be unique for particular specialties regardless of country, many aspects of work as a medical doctor are inseparably intertwined with cultural and socioeconomic conditions found in particular communities. What is more, these conditions change over time.

Furthermore, it is contentious if accepting environmental determinism is beneficial to an organization. Creating a homogenous group, as Schneider [2] indicates, may decrease the capability to adapt to the changing demands and requirements of society.

### Conclusions

To conclude, the findings of this study showed that there is a link between one’s personality and their choice of medical specialty. In general, women exhibited a greater preference for pediatric specialties, while men tended to favor surgical specialties. Introverted, Observant, and Judging individuals showed a notable inclination toward Family Medicine, while Intuitive and Prospective traits were associated with an interest in Psychiatry, particularly in Child and Youth Psychiatry. Additionally, the Thinking trait was prevalent among students opting for surgical specialties such as Neurosurgery, Forensic Medicine, Plastic Surgery, and General Surgery, whereas the Feeling trait was more common among those choosing pediatric specialties. Taking into consideration students’ personality traits, the medical curriculum should be designed to match clinical communication skills, interprofessional collaboration, and teamwork for the best outcomes of treatment, as well as physicians’ professional fulfillment.

## Appendix

Multimedia Appendix 1 16Personalities report – an analysis of each respondent's personality type and the intensity of specific personality traits.

Multimedia Appendix 2 Personality types and personality traits identified by Myers-Briggs Type Indicator (MBTI).

## Abbreviations

ASA: attraction-selection-attrition
E-I: Extroversion-Introversion
ENFJ: Extroverted, Intuitive, Feeling, Judging
ENFJ-T: Extroverted, Intuitive, Feeling, Judging, Turbulent
ENFP: Extroverted, Intuitive, Feeling, Prospecting
ENFP-T: Extroverted, Intuitive, Feeling, Prospecting, Turbulent
ESFJ: Extroverted, Observant, Feeling, Judging
ESFJ-A: Extroverted, Observant, Feeling, Judging, Assertive
ESTJ-A: Extraverted, Observant, Thinking, Judging, Assertive
ESTP: Extroverted, Observant, Thinking, Prospecting
ICD-11: International Statistical Classification of Diseases and Related Health Problems, 11th Revision
INFJ: Introverted, Intuitive, Feeling, Judging
INFJ-T: Introverted, Intuitive, Feeling, Judging, Turbulent
INTJ: Introverted, Intuitive, Thinking, Judging
INTJ-A: Introverted, Intuitive, Thinking, Judging, Assertive
INTP: Introverted, Intuitive, Thinking, Prospecting
ISFJ: Introverted, Observant, Feeling, Judging
ISFJ-T: Introverted, Observant, Feeling, Judging, Turbulent
ISFP-A: Introverted, Observant, Feeling, Prospecting, Assertive
ISTJ-A: Introverted, Observant, Thinking, Judging, Assertive
ISTP: Introverted, Observant, Thinking, Prospecting
MBTI: Myers-Briggs Type Indicator
OR: odds ratio
RIASEC: Holland’s Theory of Career Choice
STJ: Sensing, Thinking, and Judging
T-F: Thinking-Feeling

## Data Availability

The datasets generated during and analyzed during this study are available from the corresponding author upon reasonable request.
