# Supplementary material for: Personality Types of Medical Students in Terms of Their Choice of Medical Specialty: Cross-Sectional Study
Source: Interact J Med Res. 2024 Dec 31;13:e60223. doi: 10.2196/60223 (PMC11733523; doi:10.2196/60223)
Supplement: Multimedia Appendix 2 [file ijmr_v13i1e60223_app2.docx]

| **Personality type** | **Personality traits** |
| --- | --- |
| INTJ-A | Introverted, Intuitive, Thinking, Judging, Assertive |
| INTJ-T | Introverted, Intuitive, Thinking, Judging, Turbulent |
| INTP-A | Introverted, Intuitive, Thinking, Prospecting, Assertive |
| INTP-T | Introverted, Intuitive, Thinking, Prospecting, Turbulent |
| ENTJ-A | Extraverted, Intuitive, Thinking, Judging, Assertive |
| ENTJ-T | Extraverted, Intuitive, Thinking, Judging, Turbulent |
| ENTP-A | Extraverted, Intuitive, Thinking, Prospecting, Assertive |
| ENTP-T | Extraverted, Intuitive, Thinking, Prospecting, Turbulent |
| INFJ-A | Introverted, Intuitive, Feeling, Judging, Assertive |
| INFJ-T | Introverted, Intuitive, Feeling, Judging, Turbulent |
| INFP-A | Introverted, Intuitive, Feeling, Prospecting, Assertive |
| INFP-T | Introverted, Intuitive, Feeling, Prospecting, Turbulent |
| ENFJ-A | Extraverted, Intuitive, Feeling, Judging, Assertive |
| ENFJ-T | Extraverted, Intuitive, Feeling, Judging, Turbulent |
| ENFP-A | Extraverted, Intuitive, Feeling, Prospecting, Assertive |
| ENFP-T | Extraverted, Intuitive, Feeling, Prospecting, Turbulent |
| ISTJ-A | Introverted, Observant, Thinking, Judging, Assertive |
| ISTJ-T | Introverted, Observant, Thinking, Judging, Turbulent |
| ISFJ-A | Introverted, Observant, Feeling, Judging, Assertive |
| ISFJ-T | Introverted, Observant, Feeling, Judging, Turbulent |
| ESTJ-A | Extraverted, Observant, Thinking, Judging, Assertive |
| ESTJ-T | Extraverted, Observant, Thinking, Judging, Turbulent |
| ESFJ-A | Extraverted, Observant, Feeling, Judging, Assertive |
| ESFJ-T | Extraverted, Observant, Feeling, Judging, Turbulent |
| ISTP-A | Introverted, Observant, Thinking, Prospecting, Assertive |
| ISTP-T | Introverted, Observant, Thinking, Prospecting, Turbulent |
| ISFP-A | Introverted, Observant, Feeling, Prospecting, Assertive |
| ISFP-T | Introverted, Observant, Feeling, Prospecting, Turbulent |
| ESTP-A | Extraverted, Observant, Thinking, Prospecting, Assertive |
| ESTP-T | Extraverted, Observant, Thinking, Prospecting, Turbulent |
| ESFP-A | Extraverted, Observant, Feeling, Prospecting, Assertive |
| ESFP-T | Extraverted, Observant, Feeling, Prospecting, Turbulent |
